# Development of HFD‐Fed/Low‐Dose STZ‐Treated Female Sprague‐Dawley Rat Model to Investigate Diabetic Bone Fragility at Different Organization Levels

**DOI:** 10.1002/jbm4.10379

**Published:** 2020-10-20

**Authors:** Praveer Sihota, Ram Naresh Yadav, Sumathi Poleboina, Vishwajeet Mehandia, Sanjay Kumar Bhadada, Kulbhushan Tikoo, Navin Kumar

**Affiliations:** ^1^ Department of Mechanical Engineering Indian Institute of Technology Ropar Rupnagar India; ^2^ Department of Pharmacology and Toxicology National Institute of Pharmaceutical Education and Research Mohali India; ^3^ Department of Endocrinology Post Graduate Institute of Medical Education and Research Chandigarh India

**Keywords:** BONE, COLLAGEN CROSS‐LINKS, CRYSTALLITE SIZE, MATERIAL PROPERTY, NONOBESE RAT MODEL, TYPE 2 DIABETES

## Abstract

Type 2 diabetes (T2D) adversely affects the normal functioning, intrinsic material properties, and structural integrity of many tissues, and bone fragility is one of them. To simulate human T2D and to investigate diabetic bone fragility, many rodent diabetic models have been developed. Still, an outbred genetically normal nonobese diabetic rat model is not available that can better simulate the disease characteristics of nonobese T2D patients, who have a high prevalence in Asia. In this study, we used a combination treatment of high‐fat diet (4 weeks, 58% kcal as fat) and low‐dose streptozotocin (STZ; 35 mg/kg i.p. at the end of the fourth week) to develop T2D in female Sprague‐Dawley (SD) rats. After 8 weeks of the establishment of the T2D model, the femoral bones were excised after euthanizing rats (animal age approximately 21 to 22 weeks; *n* = 10 with T2D, *n* = 10 without diabetes). The bone microstructure (μCT), mechanical, and material properties (three‐point bending, cyclic reference point indentation, nanoindentation), mean mineral crystallite size (XRD), bone composition (mineral‐to‐matrix ratio, nonenzymatic cross‐link ratio [NE‐xLR], Fourier transform‐infrared microspectroscopy), and total fluorescent advanced glycation end products were analyzed. We found that diabetic bone had reduced whole‐bone strength and compromised structural properties (μCT). The NE‐xLRs were elevated in the T2D group, and strongly and negatively correlated with postyield displacement, which suggests bone fragility was caused by a lack of glycation control. Along with that, the decreased mineral‐to‐matrix ratio and modulus, increased indentation distance increase, and wider mineral crystallite size in the T2D group were evidence that the diabetic bone composition and material properties had changed, and bone became weaker with a tendency to easily fracture. Altogether, this model simulates the natural history and metabolic characteristics of late‐stage T2D (insulin resistance and as disease progress develops, hypoinsulinemia) for nonobese young (and/or adolescent) T2D patients (Asians) and provides potential evidence of diabetic bone fragility at various organization levels. © 2020 The Authors. *JBMR Plus* published by Wiley Periodicals, Inc. on behalf of American Society for Bone and Mineral Research.

## Introduction

Type 2 diabetes (T2D) is characterized by high blood glucose levels resulting from insulin resistance and/or relative insulin deficiency.^(^ ^1^ ^)^ It is a disease that causes a substantial socioeconomic burden globally.^(^ ^2^ ^)^ Diabetes potentially affects almost every organ in the human body and causes head‐to‐toe damage: heart, kidney, nerves, eye, skin, blood vessels, and bone.^(^ ^3^ ^)^ Nearly 50% to 80% increased extremity fracture risk has been observed in people with diabetes.^(^ ^4^ ^)^ Furthermore, approximately 60% of the world's diabetics live in Asia, which has the fastest population growth rate; hence, this statistic is expected to increase further in the coming years.^(^ ^5^ ^)^

In addition to the burden of disease in Asia, the phenotypes of Asian type 2 diabetics are also distinct as compared with non‐Asians (particularly Caucasians) because Caucasian diabetics tend to be obese, whereas a large proportion of patients with T2D in Asian countries are nonobese.^(^ ^6^, ^7^, ^8^, ^9^, ^10^ ^)^ At present, to simulate human T2D and to investigate the relationships of T2D and bone, studies of both obese and nonobese rodent diabetic models—based on spontaneous, monogenic abnormal leptin/leptin receptor signaling, and diet‐induced obesity—have reported weaker diabetic bones.^(^ ^11^, ^12^, ^13^, ^14^, ^15^, ^16^, ^17^, ^18^, ^19^, ^20^, ^21^, ^22^, ^23^ ^)^ For example, Saito and colleagues^(^ ^20^ ^)^ and Zhang and colleagues^(^ ^11^ ^)^ used a spontaneously nonobese diabetic rat model in their studies. However, in their models, the development of diabetes was highly genetically determined, unlike the heterogeneity seen in humans. In addition, in humans, T2D is multifactorial and strongly associated with lifestyle and dietary factors.^(^ ^15^ ^)^ In Zucker fatty rats and Zucker diabetic fatty (ZDF) rats, severe obesity develops based on hyperphagia caused by abnormal leptin/leptin receptor signaling. But leptin or leptin receptor deficiency is not an important contributor to the development of diabetes in humans (vary rare <1%).^(^ ^16^, ^21^, ^22^, ^23^ ^)^ On the other hand, the Zucker diabetic Sprague‐Dawley (ZDSD; develop diabetes over time based on polygenetic and environmental factors, as well as dietary manipulation) and UC Davis type 2 diabetes mellitus (UCD‐T2DM) rats better simulate the development and progression of T2D for Westernized societies (white population), where a high rate of T2D is caused by dietary‐induced obesity.^(^ ^16^, ^22^, ^24^ ^)^ Furthermore, in diet‐induced obesity models, the feeding of a high‐fat diet (HFD) alone requires a long time and in that too no hyperglycemia develops upon simple dietary treatment in genetically normal animals.^(^ ^25^ ^)^ Hence, there is a need to establish an ideal rat model for T2D by using outbred genetically normal rats, which can better simulate the natural history and metabolic characteristics of nonobese type 2 diabetic patients (mainly Asians). Also, it can be used to characterize the skeletal fragility in nonobese type 2 diabetics.

Here, we hypothesized that the T2D disease pattern can be achieved by combining a HFD and a low‐dose streptozotocin (STZ) treatment, designed to cause mild hyperglycemia (a condition similar to prediabetes) based on insulin resistance (because of the feeding of a HFD for 4 weeks, hyperinsulinemia). By developing a model, we could characterize the bone composition, material properties, and structural properties of diabetic bone. Hence, we developed a HFD‐fed and low‐dose STZ‐treated T2D rat model by using genetically normal outbred female SD rats that simulate the natural history and metabolic characteristics of the nonobese young (and/or adolescent) Asian T2D patients. We also further investigated the skeletal fragility parameters: structural and mechanical properties of the femoral bone, bone material properties (cyclic reference point indentation [cRPI], nanoindentation [NI]), mean crystallite size (XRD), collagen cross‐links (nonenzymatic cross‐links [NE‐xL]), and fluorescent advanced glycation end products (fAGEs) content in diabetic femoral cortical bone as compared with the controls. This study provides extensive evidence of and detailed insight into the effects of T2D on the cortical bone quality of nonobese genetically normal outbred female SD rats.

## Material and Methods

### Animals

Approximately 7‐ to 8‐week‐old female SD rats (170 to 180 g) were procured from the central animal facility of the National Institute of Pharmaceutical Education and Research, S.A.S. Nagar, Mohali at the beginning of the study. They were maintained under standard environmental conditions: temperature 20 ± 2°C, humidity 50% ± 10%, and 12‐hour light/dark cycle with food and water *ad libitum*. All protocols were approved by the Institutional Animal Ethics Committee (IAEC Approval Number 17/74, NIPER) and performed in accordance with the guidelines of the Committee for the Purpose of Control and Supervision of Experiments on Animals (CPCSEA), New Delhi, India.

### Experimental design

Animals were acclimatized for 1 week before initiation of the experiment. Then, the animals were divided into two groups. The control group was fed with commercially available normal pellet diet (12% kcal as fat; Pranav Agro Industries, New Delhi, India); the diabetic group received a HFD (in‐house prepared round balls, 58% kcal of fat) for 4 weeks, which causes insulin resistance in animals. At the end of the fourth week, they were injected with a low dose of STZ (Sigma‐Aldrich, St. Louis, MO, USA): 35 mg/kg dissolved in ice‐cold 0.01M citrate buffer, pH 4.4 i.p., after fasting for 12 hours. Control animals received an equivalent volume of vehicle (0.9% saline solution). One week after the injection of STZ, plasma glucose levels were measured, and rats showing a blood glucose concentration of more than 250 mg/dL were included in the T2D group. The rats were allowed to continue to feed on their respective diets until the end of the study.^(^ ^23^ ^)^ The diabetic group rats became insulin‐resistant because of their HFD; hence, even a slight insult by a low dose of STZ would compromise β‐cell function and lead to hypoinsulinemia.

**Figure 1:**
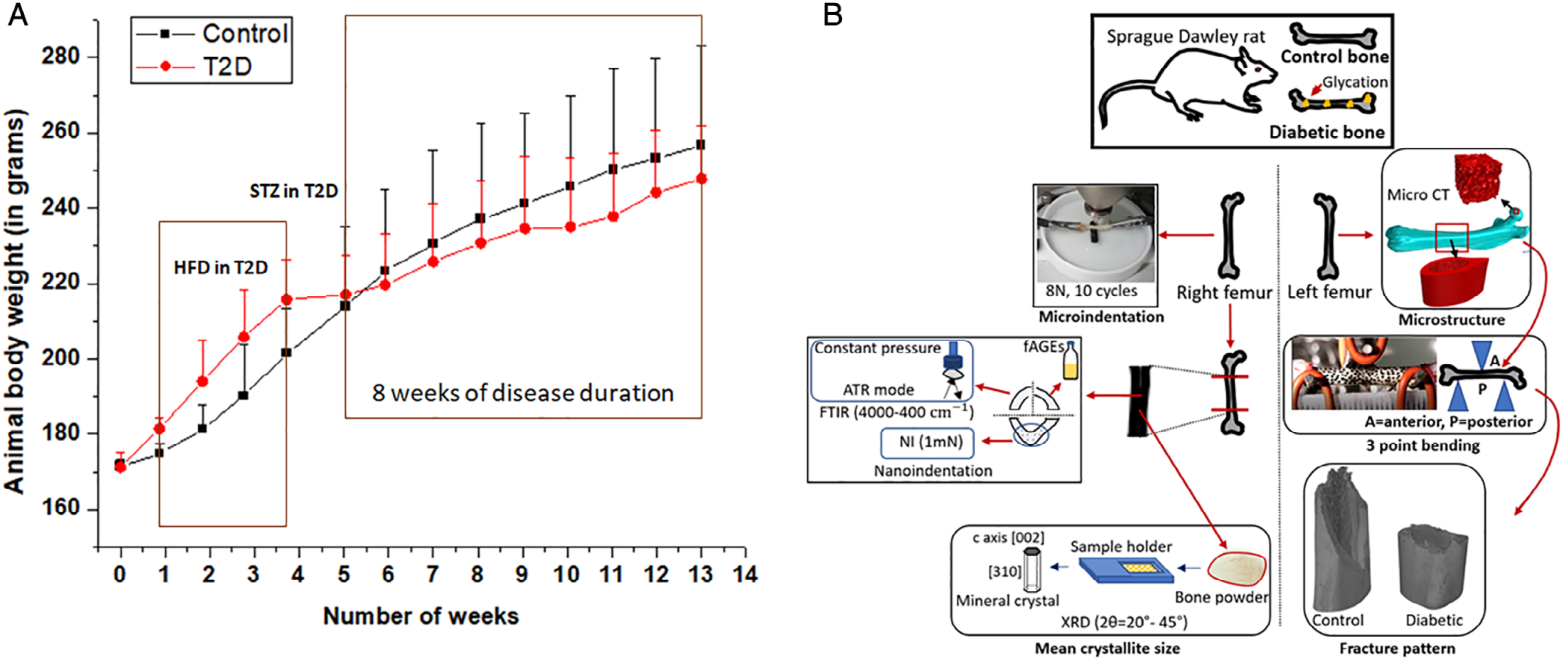
(*A*) Body‐weight changes in HFD‐fed/low‐dose STZ treated female SD rats compared with normal pellet‐diet‐fed rats by week, from the beginning to the end of the study. (*B*) Schematic of materials and methods and allocation of tissue for each characterization technique. Left femora were used for microstructural (μCT), mechanical (three‐point bending), and fracture pattern analysis, and right femora were utilized to determine properties at the material level (cRPI), bone composition with FTIR, mean crystal size with XRD, and fAGEs content. cRPI = cyclic reference point indentation; fAGE = fluorescent advanced glycation end products; FTIR = Fourier transform‐infrared microspectroscopy; HFD = high‐fat diet; SD = Sprague‐Dawley; STZ = streptozotocin; T2D = type 2 diabetes; XRD = mean crystallite size.

Body weight was recorded every week, from the beginning to the end of the study (Fig. 1*A*). At the end of the study, overnight‐fasted rats were anesthetized under light ether, and blood samples were collected from the tip of the tail vein. Blood samples were collected in EDTA for determination of HbA1C and the preparation of plasma. Plasma glucose and blood HbA1c were estimated as per the manufacturer's guidelines (Accurex Biomedical Pvt. Ltd., Mumbai, India; Tulip Diagnostics Pvt. Ltd., Goa, India).

After euthanization, the femora from both groups were dissected, wrapped in PBS‐soaked gauze, kept in zip‐locked plastic bags, labeled, and subsequently stored at −80°C. Before the experiments, the bones were equilibrated to room temperature. The left femora were utilized for microstructural (μCT), three‐point bending, and fracture‐pattern analysis; the right femora were used to determine properties at the material level (cRPI and NI), bone composition with Fourier transform‐infrared microspectroscopy (FTIR) and fAGEs content as shown in Fig. 1*B*.

### Measurement of bone quality parameters

#### 1 *Structural parameters*

To determine the structural parameters of cortical (mid‐diaphysis of femoral) and trabecular (femoral head) bone, the left femora were scanned along the cylindrical axis using the nanotom‐S nano‐CT system (Phoenix/X‐ray; GE Sensing & Inspection Technologies, Hurth, Germany). The source was set at 55 kV and 160 μA, exposure time 500 ms, frame averaging 7, and a voxel size of 20 μm and 5 μm to scan the entire femoral bone and femora head, respectively. For bone‐density calibration, two phantoms with 200 mg/cc and 800 mg/cc of hydroxyapatite (QRM GmbH, Mohrendorf, Germany) were imaged with the same parameters as the bone samples. Later, the mean gray value was calibrated based on the linear relationship between gray value and density.^(^ ^26^ ^)^

The reconstruction of raw data was performed using Datos.rec (phoenix/X‐ray; GE Measurement & Control, Wunstorf, Germany). The bone was segmented from background based on the grayscale value of the image, and for segmentation manual thresholding was done (with suitable gray scale values of trabecular and cortical bone separately). A noise removal Gaussian filter (*σ* = 1) was applied, and then reconstructed images were imported in Scan‐IP (Simpleware Ltd, Exeter, UK) and ImageJ's plugin BoneJ^(^ ^27^ ^)^ (NIH, Bethesda, MD, USA; https://imagej.nih.gov/ij/). The midpoint between the greater trochanter and the endpoint of the distal femur was calculated to identify mid‐diaphysis. Then, the cylindrical volume of 1 mm above and below the mid‐diaphysis was selected as the ROI to obtain a 3D model, which was utilized to calculate the microstructural parameters of the cortical bone. Likewise, the cubical volume of 1.5*1.5*1.5 mm^3^ was selected as the ROI from the center of the femoral head, to calculate the trabecular bone microstructural parameters. The following microstructural parameters were calculated: trabecular volume fraction (BV/TV; %), trabecular number (Tb.N; 1/mm), trabecular thickness (Tb.Th; mm), trabecular separation (Tb.Sp; mm), structure model index (SMI), degree of anisotropy (DA), connectivity density (Conn.D), cortical bone area (Ct.Ar; mm^2^), average cortical thickness (Ct.Th; mm), and polar moment of inertia (J; mm^4^). This study was performed according to a previously published protocol.^(^ ^28^ ^)^

### Material properties

#### 1 *Three‐point bending test*

After μCT imaging, the left femora were utilized for the three‐point bending test. The femora were kept in saline‐soaked gauze until immediately before testing. The bones were placed in such a way that the posterior side down on the bottom support (19‐mm span) and the central loading roller applied force at mid‐diaphysis, as described in a previously published protocol.^(^ ^16^ ^)^ The radius of curvature of each support was 2 mm. The specimens were preloaded with 10 N to ensure proper contact between the test specimen and the loading roller. To test samples until failure, a single‐cycle ramp function at a constant displacement rate of 10 mm/min was applied with no displacement end limit.^(^ ^29^, ^30^ ^)^ The bending tests were performed using an electromagnetic testing system (Electroforce 3200; Bose, Eden Prairie, MN, USA) at room temperature while keeping the specimen hydrated via a PBS spray. To ensure uniform deformation without any movement of the specimen, a digital microscope (Dino‐Lite 5MP, Taiwan) was utilized. The load–displacement data were captured at a 0.01‐s time interval, and were further used to calculate the maximum load (F_max_, N), stiffness (N/mm), work‐to‐failure (N/mm), and postyield displacement (PYD; mm). Fmax was the greatest load achieved before fracture; stiffness was measured as the slope of the linear portion of the load–displacement curve; work‐to‐failure was represented as the area under the load–displacement curve (whole‐bone toughness). PYD referred to displacement (D) that occurs between yielding (D_yield_) and fracture (D_fx_) [PYD = D_fx_ – D_yield_]. The yield point was calculated with a 0.2% offset method.^(^ ^31^ ^)^

#### 2 *Cyclic reference point indentation*

The mid‐diaphysis region of right femora was tested by a cRPI instrument under a wet condition. It provided a measure of bone mechanical properties, in particular, the resistance of the bone to microindentation at the tissue level. Six indents were performed on the anterior region of femora, separated by 1 to 2 mm, repeatedly for 10 indentation cycles at a frequency of 2 Hz, with a maximum force of 8 N. Before actual measurement, the probes were tested on a poly(methyl methacrylate) block according to the manufacturer's instructions to ensure proper function. Later, for fixed load, the distance by which the probe inserted into the bone was recorded, which was used to calculate indentation distance increase (IDI; μm), average energy dissipation (Avg‐ED; μJ), first cycle unloading slope (US‐1st, N/μm) and total indentation distance (TID, μm). Measurements were averaged for each sample and used to calculate the mean of each group. Testing was done according to a previously published protocol.^(^ ^32^ ^)^

The IDI with cyclic loading to a fixed force is an important parameter of cRPI to distinguish the fragile bone from less easily fractured bone.^(^ ^33^ ^)^ It is the absolute penetration depth increase from the first cycle to the last cycle of each test, and it is inversely correlated to the bone toughness.^(^ ^34^ ^)^ Other outcomes of interest are the US‐1st (slope of the unloading portion of the first cycle), an indicator of bone material stiffness, and Avg‐ED as a measure of unrecovered bone deformation (plasticity).

#### 3 *Nanoindentation*

The cross‐section of mid‐diaphysis of right femora was cut with a low‐speed diamond blade saw (IsoMet; Buehler, Lake Bluff, IL, USA), and then embedded in epoxy, which takes nearly 2 hours to get cured. After curing, the samples were ground (Buehler Eco Met 250 grinder and polisher) with abrasive papers of 1200 and 2000 grit size under the water‐cooling condition and polished with diamond solutions of particle sizes of 1, 0.5, and 0.25 μm, and then samples were sonicated for 10 min. The NI experiment was carried out within an hour using a TI‐950 Tribo Indenter (Hysitron Inc., Minneapolis, MN, USA) with a Berkovich pyramidal tip in the moist state. Eight indents with a peak load of 1000 μN were applied to the cross‐section of the bone. A load function consisting of a 10‐s loading and unloading segment and a 10‐s hold time. The load–displacement curves obtained in these indentation tests were analyzed to determine the reduced modulus (*E*_*r*_) and hardness (*H*) by using the method of Oliver and Pharr.^(^ ^35^ ^)^

### Biochemical analysis

**Figure 2:**
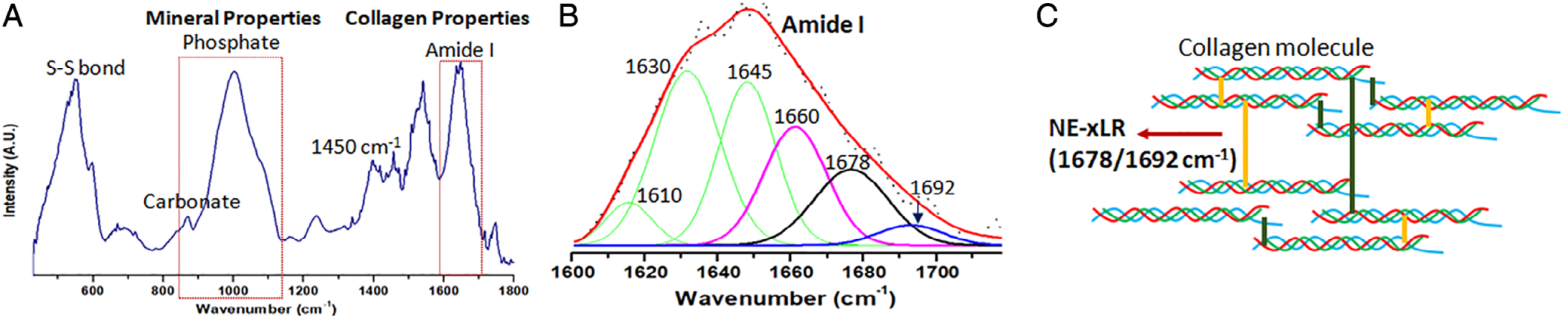
(*A*) Representative Fourier transform‐infrared microspectroscopy spectra with appropriate label of various bands to analyze the type 2 diabetic (T2D) and control femoral cortical bone. (*B*) Peak‐fitting of amide I band. Collagen properties were obtained by peak‐fitting of amide I band with sub‐bands (Gaussian curves) at 1610, 1630, 1645, 1660, 1678, and 1692 cm^−1^. (*C*) Schematic of the nonenzymatic cross‐link (NE‐xL) formation in bone collagen.

The one‐fourth diaphysis of bone samples was freeze‐dried overnight and directly broken into a particle size of a few microns by using a mortar and pestle. Later, the FTIR spectra were recorded with the help of Bruker IFS 66v/S FTIR spectrophotometer in attenuated total reflectance mode under constant pressure in the spectral region of 1800 to 400 cm^−1^ as shown in Fig. 2*A*. After recording the spectra, OriginPro 8 (OriginLab, Northampton, MA, USA) software was used to do baseline correction, and calculate peak intensity, as well as area under the curve. The mean values were also calculated for each measured parameter of each group.

The following bone mineral‐ and collagen‐related parameters were calculated. The mineral parameters included the mineral‐to‐matrix ratio (area ratio of the phosphate ν1 to ν3 peak [916 to 1180 cm^−1^] to amide I peak [1596 to 1712 cm^−1^]), the carbonate‐to‐phosphate ratio (area ratio of the carbonate ν2 peak [852 to 890 cm^−1^] to the phosphate ν1 to ν3 peak [916 to 1180 cm^−1^]), the mineral crystallinity (intensity ratio of 1030 to 1020 cm^−1^, which is related to crystal size and stoichiometric perfection), and the acid phosphate content (intensity ratio of 1127 to 1096 cm^−1^, which characterizes acid phosphate substitution into stoichiometric hydroxyapatite).^(^ ^36^, ^37^ ^)^

The collagen parameters were measured through the amide I peak because it possesses structural information about the collagen matrix and is also the location of the strongest peaks for the nonenzymatic cross‐link (NE‐xL) pentosidine (AGE).^(^ ^38^ ^)^ Thus, sub‐bands of the amide I band were peak‐fit with Gaussian curves at 1610, 1630, 1645, 1660, 1678, and 1692 cm^−1^ by using the peak analyzer tool in OriginPro 8.5 software as shown in Fig. 2*B*. These peaks were chosen based on second‐derivative spectra. From the analysis of amide I sub‐bands, the nonenzymatic cross‐link ratio (E‐xLR, area ratio of 1678 to 1692 cm^−1^ sub‐bands) and the collagen maturity (area ratio of 1660 to 1690 cm^−1^ [related to the ratio of pyridinoline to divalent cross‐links]) were measured within the amide I peak, where the measurement of NE‐xLR enables the measurement the collagen quality associated with NE‐xL and is an indirect measure of overall AGEs (which make cross‐links) content in bone tissue itself.^(^ ^38^, ^39^ ^)^ The schematic presentation of NE‐xL is shown in Fig. 2*C*.

#### 1 *Mean crystallite size*

The bone samples of the right femora were powdered directly using a mortar and pestle by keeping in acetone. The bone powder was dried in a fume hood and transferred in tightly sealed cryovials. The Panalytical X'Pert Pro multipurpose diffractometer (PANalytical, Almelo, The Netherlands) was used to record XRD patterns with 40 kV and 40 mA, with no spinning. Cu‐tube by CuKα radiation wavelength of 1.5406 A° was used. The experiments were conducted with the slow scan at 2θ = 20° to 45°, with a step size of 0.0334°/2θ and count time at each step of 250 s. The X'pert Highscore plus software was used for postprocessing, to do background correction and fit the diffraction peaks observed at 2θ = 26° (belongs to the c‐axis direction, [002 plane] indicates the average length of crystal) and 2θ = 40° (belongs to [310 plane] perpendicular to the c‐axis direction, indicates the average width of crystal).^(^ ^40^ ^)^ The average crystallite size of bone mineral was obtained by using the Scherrer equation, B(2θ) = λ/LCosθ, where B is the mean crystallite size, λ is the X‐ray wavelength, θ is the Bragg angle, and L is the peak width at half‐maximum. The protocol was adopted from published studies.^(^ ^40^, ^41^ ^)^

#### 2 *AGE* assay

Total fAGEs were measured using fluorescence spectrometry and normalized to collagen concentration as in previous studies.^(^ ^42^, ^43^, ^44^ ^)^ The cortical bone samples were lyophilized overnight then hydrolyzed in 6 N HCl (100 μL/mg bone) at 110°C for 20 hours in hydrolysis vials with screw caps. The hydrolysate was cooled at room temperature, collected in a microcentrifuge tube, and centrifuged with 13000 rpm at 4°C (Eppendorf 5424R microcentrifuge, Hamburg, Germany). The supernatant was collected and diluted (10 times with DI water); fluorescence was measured in a flat‐bottom 96‐well plate using a multimode microplate reader (CLARIOstar Plus; BMG LABTECH, Ortenberg, Germany) at an excitation of 360 nm and an emission of 460 nm. The fluorescence of the bone samples was normalized with serially diluted quinine standards (stock: 10 μg quinine per 1 mL of 0.1 N H_2_SO_4_) measured in the same way.

Next, to quantify hydroxyproline (absorbance assay), a chloramine‐T solution (0.05M chloramine T, 2‐methoxyethanol and hydroxyproline buffer in 2:3:5, respectively) was added to the hydroxyproline standards (stock solution: 2 mg hydroxyproline in 1 mL 0.001 N HCl) and the bone hydrolysates (as mentioned above) in 1:2 ratio and incubated for 20 min at room temperature in the dark to oxidize hydroxyproline, then 3.15M perchloric acid was added to the above solution and incubated for 5 min to quench chloramine‐T. At last, the *p‐*dimethylaminobenzaldehyde solution was added, and the mixture was incubated for 20 min at 60°C, a color change was observed in this incubation, then samples were cooled at room temperature for 5 min, and absorbance of the specimens and standards was measured at a wavelength of 570 nm using the same microplate reader. Total fAGEs are reported in units of ng quinine fluorescence/mg collagen. The collagen content was derived based on prior knowledge that collagen consists of 14% hydroxyproline.^(^ ^45^ ^)^ All experiments were performed in darkness at room temperature.

### Statistical analysis

Distributions for all variables were plotted to identify potential outliers, and the points beyond 2 SDs of the mean were removed from the analysis. Statistical analysis was performed using SPSS (v.21; SPSS Inc., Chicago, IL, USA) and Microsoft Office Excel (2007). The distribution of the data was tested for normality by the Kolmogorov–Smirnov test. Homogeneity of variances was analyzed using Levene's test. Between‐group differences of calculated parameters were analyzed for statistical significance using Student's *t* tests or Mann–Whitney *U* tests, as appropriate, after testing for normality and homogeneity of variances. The mean values and SDs were calculated for the measured parameters. Pearson correlation tests were used to determine relationships between variables in the T2D and control groups separately. Forward stepwise regression tests were conducted for mechanical properties using measures of glycation (fAGE and NE‐xLR) as independent variables. A confidence level of *p* < 0.05 implies a statistical significance between the groups, where *p* < 0.05, *p* < 0.01, and *p* < 0.001 denote the level of significance.

## Results

After 4 weeks of HFD‐feed, the plasma insulin value was higher from 1.72 ± 0.058 to 2.61 ± 0.088 mIU/L (*p* < 0.001) in the control to the T2D groups, respectively, which evidenced the condition of hyperinsulinemia caused by insulin resistance. After 8 weeks of the establishment of the T2D model (Table 1), the difference in body weight was nonsignificant, the difference in fasting blood glucose and HbA1c was found statistically higher (*p* < 0.001) in the T2D group as compared with the control group. The plasma insulin value was decreased by 21.4% (*p* = 0.038) in the T2D group as compared with the control group, which evidenced the condition of hypoinsulinemia. Also, the T2D group animals showed the abnormalities in lipid metabolism as evidenced by significantly increased plasma triglyceride (*p* < 0.001) and plasma total cholesterol levels (*p* = 0.001), which contribute to various cardiovascular complications.

**Table 1:** Body Weight and Blood Glucose of Control and T2D Groups at the End of the Study; and Microstructural Parameter Findings of Control and T2D Rat Femoral Bone

	Control (*n* = 10)	T2D (*n* = 10)	*p* Value
Body weight (gm)	252.8 ± 30.67	247.8 ± 13.92	0.748
Fasting glucose (mg/dL)	97.9 ± 10.46	292.5 ± 45.69	<0.001***
HbA1c (%)	6.08 ± 0.39	7.89 ± 0.52	<0.001***
Plasma insulin (mIU/L)	2.82 ± 0.588	2.216 ± 0.197	0.038*
Plasma triglyceride (mg/dL)	64.21 ± 8.39	213.66 ± 29.06	<0.001***
Plasma total cholesterol (mg/dL)	63.68 ± 7.78	130.64 ± 26.53	0.001**
Microstructural parameters (μCT)			
Trabecular bone parameters			
Trabecular volume fraction (BV/TV; %)	46.14 ± 2.10	38.54 ± 6.05	0.015*
Trabecular number (Tb.N; 1/mm)	5.048 ± 1.20	4.32 ± 0.44	0.210
Trabecular thickness (Tb.Th; mm)	0.095 ± 0.017	0.089 ± 0.011	0.539
Trabecular separation (Tb.Sp; mm)	0.142 ± 0.037	0.166 ± 0.029	0.23
Structure model index (SMI)	1.29 ± 0.68	1.51 ± 0.48	0.547
Degree of anisotropy (DA)	3.39 ± 0.59	3.87 ± 1.56	0.50
Connectivity density (Conn.D; 1/mm^3^)	775 ± 290	458 ± 251	0.43
Trabecular tissue mineral density (Tb.TMD; mg/cc)	739.0 ± 343	963.0 ± 105	0.160
Cortical bone parameters			
Cortical tissue mineral density (Ct.TMD; mg/cc)	1594.7 ± 50	1604.1 ± 114	0.87
Cortical area (Ct.Ar; mm^2^)	6.76 ± 0.28	5.72 ± 0.85	0.019*
Cortical thickness (Ct.Th; mm)	0.70 ± 0.02	0.61 ± 0.05	0.013*
Polar moment of inertia (J; mm^4^)	0.34 ± 0.046	0.26 ± 0.056	0.035*

All data are expressed as mean ± SD; *p < 0.05, **p < 0.01, ***p < 0.001, respectively, compared with the control group. HbA1c = glycosylated hemoglobin A1c; T2D = type 2 diabetes.

### Structural parameters

The mean values of microstructural parameters are calculated and shown in Table 1 for the T2D and control groups. The difference in cortical and trabecular tissue mineral density (Ct.TMD or Tb.TMD) did not reach the level of significance. The T2D group had significantly lower values of trabecular volume fraction (16.5%, *p* = 0.015), Ct.Ar (15.4%, *p* = 0.019), Ct.Th (12.9%, *p* = 0.013), and J (23.5%, *p* = 0.035) compared with the control group. The difference between the mean value of Tb.N (*p* = 0.21), Tb. Th (*p* = 0.54), Conn.D (*p* = 0.43), Tb. Sp (*p* = 0.23), SMI (*p* = 0.55), and DA (*p* = 0.50) did not reach the level of significance in the T2D group as compared with the control group. Even though the thinning of trabeculae and the trabecular number was not significantly different in the T2D group, lower values of these parameters contributed to reduced trabecular bone volume fraction (BV/TV) in the T2D group.

### Material properties

#### *Three‐point bending test*

**Figure 3:**
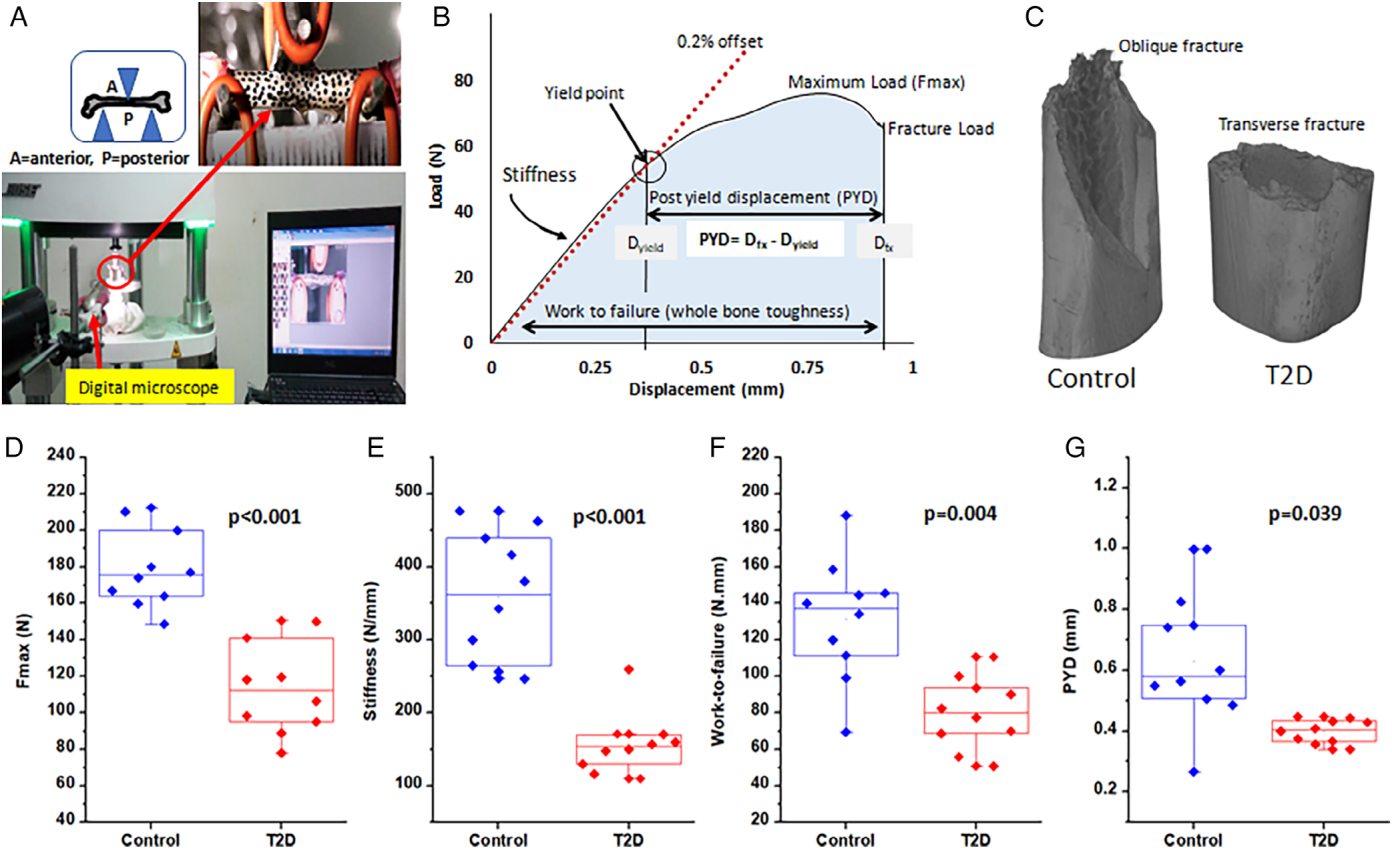
(*A*) Experimental setup of three‐point bending test. (*B*) Load–displacement curve resulting from a rat bone loaded to fracture in three‐point bending. (*C*) Image of fracture pattern obtained during three‐point bending, showing diabetic bone with a transverse fracture, whereas control bone has an oblique fracture. (*D–G*) Maximum force (Fmax), stiffness, work‐to‐failure, and postyield deflection (PYD) graphs, respectively, showing a smaller value in the type 2 diabetes (T2D) group.

The experimental setup, representative load–displacement curve, and image of fracture pattern are shown in Fig. 3*A–C,* respectively. The images of the fracture pattern for the diabetic and control groups show that the diabetic bone suffers from transverse fracture (shortest and direct crack path, *n* = 7/10), where control bone suffers from oblique fracture (increased deflection in the crack path, *n* = 9/10). This finding revealed the altered matrix properties of diabetic bone. Further, the mean values of F_max_, stiffness, work‐to‐failure, and PYD all are found to be lower by 36.9%, *p* < 0.001; 57%, *p* < 0.001; 41%, *p* = 0.004; and 36.8%, *p* = 0.039, respectively, in the diabetic group as compared with the control group as shown in Fig. 3*D–G*. Thus, diabetic bone consists of compromised load‐bearing capacity (F_max_), deformation resistance within the elastic region (stiffness), reduced capacity to absorb energy before fracture (work‐to‐failure [whole bone toughness]) and reduced ductility (PYD, plasticity) compared with the control group.

#### *Cyclic reference point indentation*

**Figure 4:**
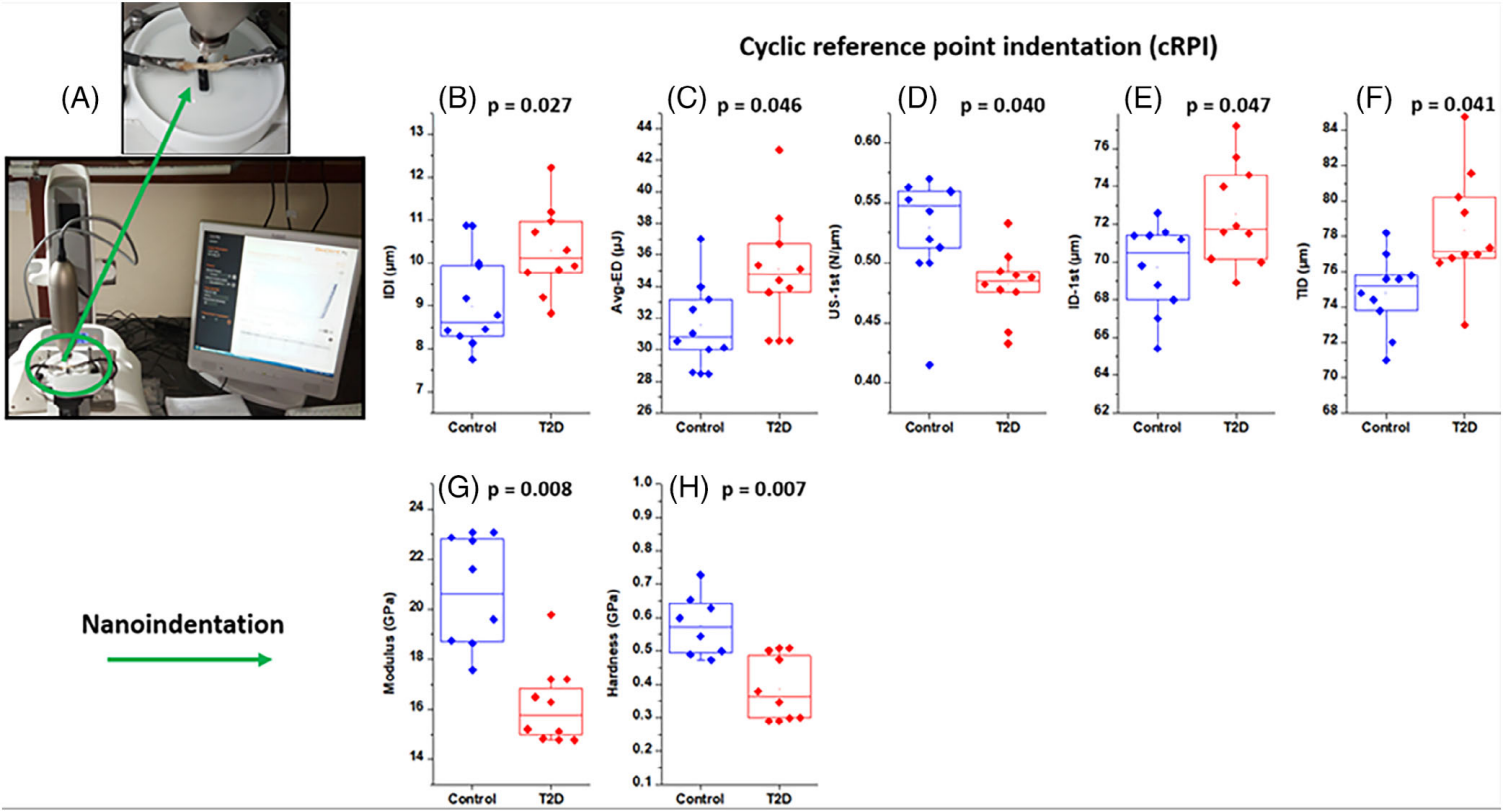
(*A*) Experimental setup of cyclic reference point indentation (cRPI) test. (*B–D*) Indentation distance increase (IDI), average energy dissipated (Avg‐ED), and unloading slop first cycle (US‐1st), respectively, showing increased value of IDI, Ave‐ED, ID‐1^st^, and total indentation distance (TID), and smaller value of US‐1st in the type 2 diabetes (T2D) group. (*E–F*) Nanoindentation results show smaller value of reduced modulus (Er) and hardness, respectively, in the T2D group.

The experimental setup and mean values of IDI, Avg‐ED, US‐1st, ID‐1st, and TID are shown in Fig. 4*A–F* for the control and T2D groups. The T2D group had significantly higher values of IDI, Avg‐ED, ID‐1st, and TID (by 14.7%, *p* = 0.027; by 11.3%, *p* = 0.046; by 4.1%, *p* = 0.047; and by 4.7%, *p* = 0.041, respectively), which indicate that the T2D bone is less resistant to fracture (IDI) and favors a larger amount of unrecovered bone deformation (Avg‐ED) as compared with controls. Also, the lower value for US‐1st (by 9.1%, *p* = 0.040) was observed in the T2D group, which indicates T2D bones have lower matrix stiffness.

#### *Nanoindentation*

The load–displacement data obtained through NI tests for both the groups reveal that under the same load of 1000 μN, the T2D group had significantly lower values of modulus (16.22 ± 0.78 GPa to 20.6 ± 1.04 GPa, *p* = 0.008) and hardness (0.387 ± 0.039 GPa to 0.577 ± 0.039 GPa**, *p* = 0.007**) because the T2D bone undergoes greater deformation, whereas control bones undergo lesser deformation under the same loading. The modulus and hardness both were found to be lower by 21.4% and 32.9%, respectively, in the T2D group as compared with the control group; the result is shown in Fig. 4*G,H*.

#### *Mean mineral crystallite size*

**Figure 5:**
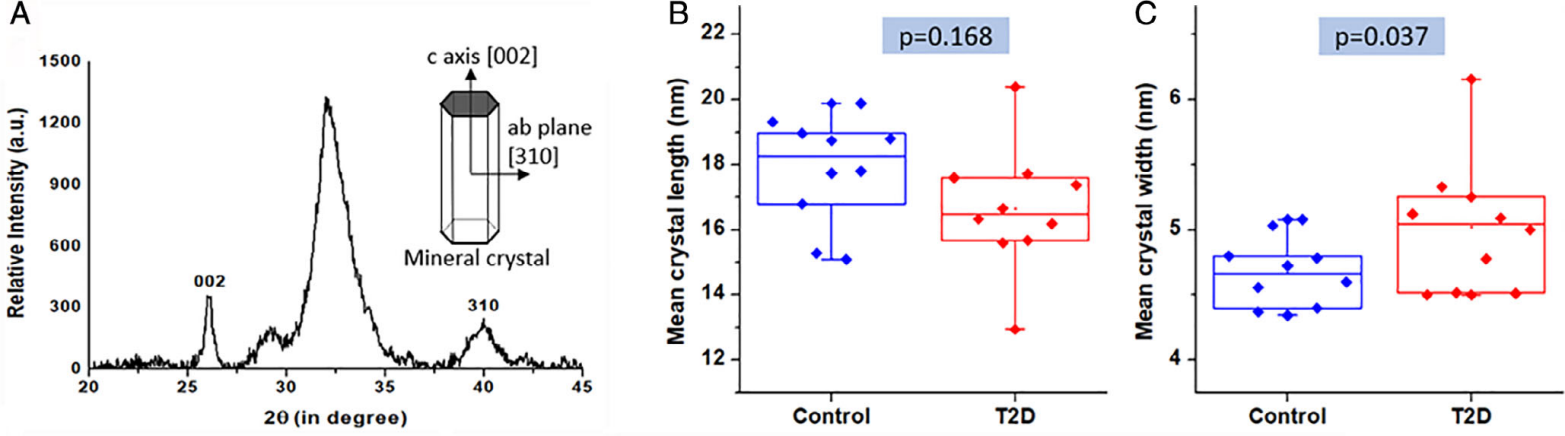
(*A*) Representative mean crystallite size (XRD) pattern (20° < 2θ < 45°) of SD rat cortical femoral bone. The peak at 26° and 40° is used to determine the average crystal length and width in the c‐axis direction [002] plane and ab‐plane [310], respectively. (*B*) Mean crystallite length and (*C*) increased width of mean crystallite in the type 2 diabetes (T2D) group.

The typical XRD pattern of cortical bone is shown in Fig. 5*A*. The average crystallite length was decreased from 18.29 ± 0.73 nm to 16.67 ± 0.85 nm, and the width was increased from 4.64 ± 0.11 nm to 5.18 ± 0.19 nm in control to T2D groups, respectively. The lower value of mean crystallite length was found insignificant (8.9%, *p* = 0.168), and crystallite width size (11.7%, *p* = 0.037) was found significant in the T2D group with respect to the control group as shown in Fig. 5*B*.

#### Compositional analysis and *fAGE* assay

**Figure 6:**
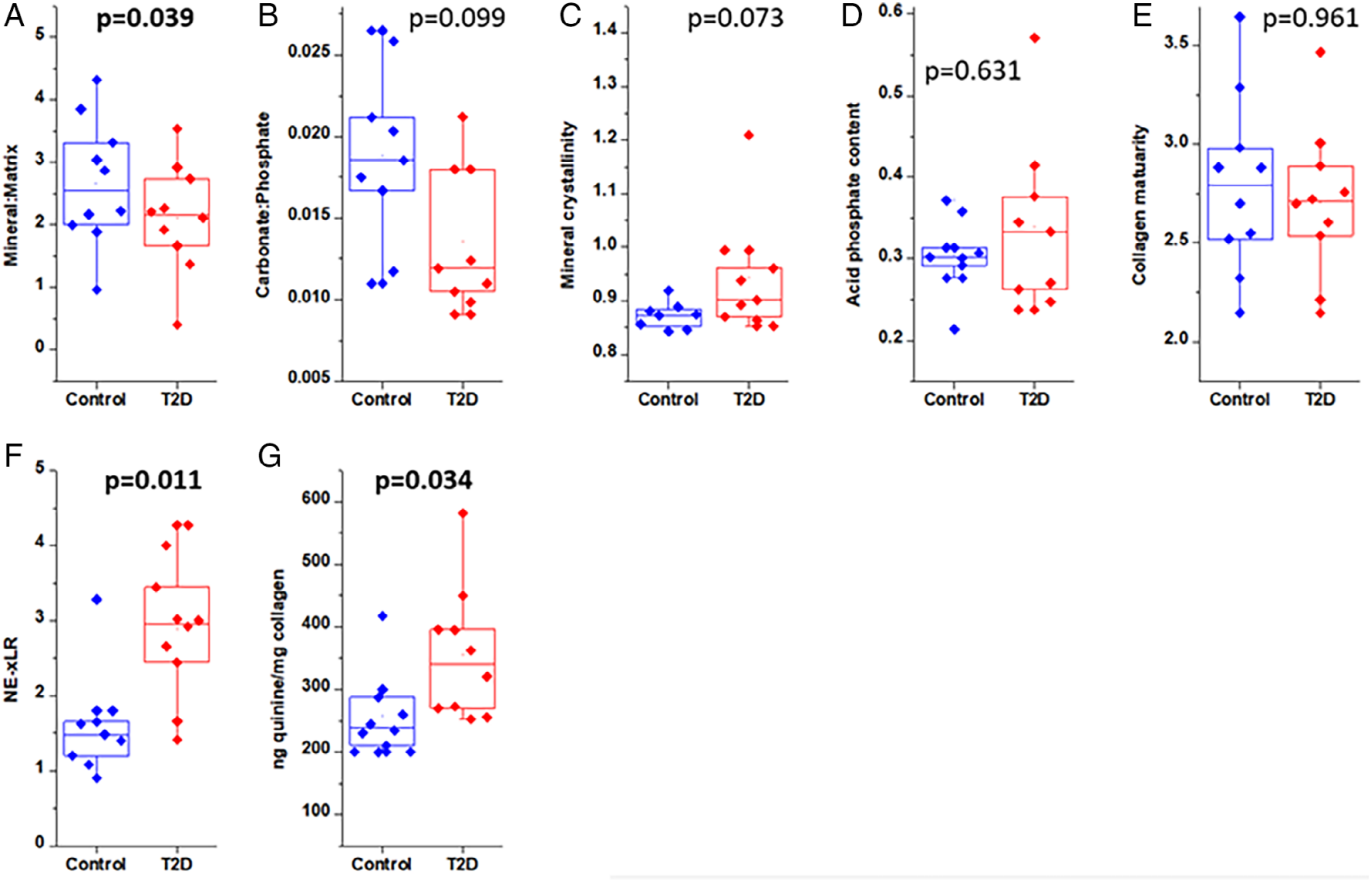
(*A–D*) Measures of mineral properties and collagen maturity, showing lower mineral‐to‐matrix ratio in type 2 diabetic (T2D) bone, whereas all other mineral parameters could not reach the level of significance. (*E*) Collagen maturity (area ratio of the 1660/1692 cm^−1^ sub‐bands). (*F*) Nonenzymatic cross‐link ratio (NE‐xLR; the area ratio of the 1678/1692 cm^−1^ sub‐bands, total cross‐linking AGEs). (*G*) The graph is showing that the fluorescent advanced glycation end products content is higher in the T2D group.

Figure 6*A–D* shows the mineral‐based parameters, where T2D bone had a lower mineral/matrix ratio (by 33.46%, *p* = 0.039), and nearly similar carbonate/phosphate ratio (by 22.22% *p* = 0.099), mineral crystallinity (by 9.93%, *p* = 0.073), and acid phosphate content (by 6.94%, 0.631) in both the groups.

The collagen parameters: collagen maturity and collagen cross‐links (NE‐xLR, area ratio of the 1678/1692 cm^−1^ sub‐bands) are calculated and shown in Fig. 6*E,F*. The T2D bone had significantly higher NE‐xLR (by 85.65%, *p* = 0.011) compared with the control bones. No change is observed in collagen maturity (*p* = 0.961).

The average fAGEs concentration was higher, from 288.6 ± 33.5 to 412.4 ± 36.6 ng quinine/mg collagen, in the control than in the T2D group, respectively, as shown in Fig. 6*G*. The fAGE concentration was found to be significantly higher (42.9%, *p* = 0.034) in the T2D group with respect to the control group, which revealed that the nonenzymatic glycation is higher in the T2D group.

#### *Interrelationship between variables*

Within the diabetic group (Supplementary Fig. S1), the HbA1c was found positively correlated with NE‐xLR **(** *r* = 0.685, *p* = 0.029), whereas the correlation between HbA1c and fAGE was found nonsignificant. HbA1c was negatively correlated with BV/TV (*r* = −0.731, *p* = 0.039), and tended to be negatively associated with cortical thickness (*r* = −0.722, *p* = 0.067) and cortical area (*r* = −0.645, *p* = 0.118) in the diabetic group. Correlations between HbA1c and mechanical parameters revealed that within the diabetic group the HbA1c was strongly and negatively correlated with F_max_ **(** *r* = −0.708, *p* = 0.033). HbA1c was also found negatively correlated with mean crystallite width (*r* = −0.752, *p* = 0.032) in the diabetic group. All other microstructural and compositional parameters were not significantly correlated with HbA1c in both groups. Also, none of the parameters in T2D, as well as the control group, was significantly correlated with plasma insulin value (lower circulating insulin) measured at the end of the study.

Within the diabetic group itself, the fAGE and NE‐xLR correlated with mean crystallite width (*r* = 0.833, *p* = 0.010) and PYD (*r* = −0.697, *p* = 0.025), respectively. However, in the nondiabetic group, the fAGE and NE‐xLR did not correlate significantly with any of the parameters.

**Figure 7:**
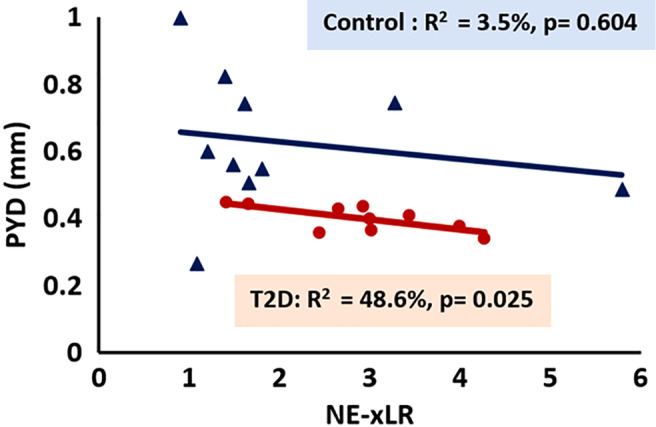
Mechanical property versus measure of glycation. Graphical data for mechanical parameter (postyield‐displacement [PYD]) versus nonenzymatic cross‐link ratio (NE‐xLR). T2D = type 2 diabetes.

Forward stepwise regression tests to predict mechanical properties as a dependent variable using measures of glycation (fAGE and NE‐xLR) as independent variables showed that within the diabetic group the NE‐xLR can explain up to 48.6% (*p* = 0.025) of variance in PYD (Fig. 7).

## Discussion

This study was initiated to develop an animal model of T2D with HFD‐fed/low‐dose STZ, by using genetically normal outbred female SD rats, which simulates the natural history and metabolic characteristics of nonobese young (and/or adolescent) T2D patients. The model would provide evidence that 8‐week‐long, persistent hyperglycemia degrades the structural, mechanical, material, and compositional properties of the femoral bone, while having comparable Ct.TMD (femur) among animals of T2D and control groups. We explored the associated changes caused by diabetes on bone quality parameters, which involves a wider mean crystallite size, decreased mineral‐to‐matrix ratio, increased nonenzymatic collagen cross‐links (NE‐xLR), and total nonenzymatic glycation content (fAGEs), and their effects on bone mechanical, microstructural, and material properties.

First, the assessment of bone microstructure after μCT showed lower cortical area (Ct.Ar) and cortical thickness (Ct.Th) in those with T2D when compared with controls. This compromised cortical microstructure results in a lower value of F_max_ in three‐point bending that indicates lower femoral bone strength in the diabetic group. Along with lower F_max_, lower values for stiffness (represents less load obtained capability to achieve a given displacement in the elastic region) and PYD (which represents the less‐ductile diabetic bone) were observed in the diabetic group. In general, ductile bone accommodates more plastic deformation before fracture, whereas brittle bone favors very little PYD.^(^ ^31^ ^)^ Likewise, a lower value for toughness was observed in the diabetic group, which shows that diabetic bone is less tough (more brittle) because the tougher bone stores a larger amount of energy before fracture.

Likewise, at the material level in the cRPI and NI tests, the lower value of the first cycle unloading slope (US‐1st, bone matrix stiffness) and reduced modulus was observed, respectively, in the diabetic group. Also, energy storage and fracture resistance were compromised in the diabetic group, as evidenced by the higher value of Avg‐ED and IDI in the diabetic bone as compared with the control group. Our finding is consistent with the literature, where higher IDI (result of cyclic test) was found to have a deeper indentation and more easily fractured bone.^(^ ^33^, ^46^, ^47^ ^)^

The lower values for modulus and hardness (NI) were directly associated with an altered bone composition, which was evidenced by the lower mineral‐to‐matrix ratio (FTIR) in the diabetic bone. In a previous study on ZDSD rats, Hammond and colleagues^(^ ^48^ ^)^ found increased values for the mineral‐to‐matrix ratio relative to controls, which might be because they used an obese diabetic rat strain. Another possible reason for decreased reduced modulus (NI) is the wider mean crystallite size (XRD) in the T2D group. Indeed, the wider crystallite size with constant length results in a decreased aspect ratio (surface area/volume) of apatite crystals, absorbs little energy, and results in reduced elastic modulus of bone material.^(^ ^49^ ^)^ Furthermore, this altered crystal shape also can affect crystal connectivity, orientation, and arrangement,^(^ ^49^ ^)^ which may results in reduced maximum loading capacity. This result is consistent with the finding of a previous study, which found that the bones of older animals and osteoporotic patients have higher crystal size and that their bone tends to fracture more easily.^(^ ^50^, ^51^ ^)^ Similar to our results, Boyar and colleagues^(^ ^52^ ^)^ also identified increased apatite crystal sizes for the diabetic femur, indicating the increased crystallinity that is widely seen in osteoporosis.^(^ ^52^ ^)^

In addition, the weaker diabetic bone behavior was evidenced by a transverse (in‐plane) fracture pattern, which indicates the relative loss of bone material heterogeneity, whereas the control bone showed diagonal (oblique/out of plane) fractures, which indicates the quasi‐brittle (heterogenous) nature of the bone material. Our finding is consistent with a previous study that reported that the failure of tougher bone specimens is governed by increased deflection and longer crack path, whereas a shorter, more‐direct crack path, as well as less‐distributed damage, were evidenced during the failure of weaker bones.^(^ ^53^ ^)^ This might be because relatively very similar elastic modulus (material level) in the bone of the T2D group could lead to small and straight crack paths compared with the control group.

We hypothesized the main reason for weaker and brittle bone in T2D is because of prolonged hyperglycemia (increased HbA1c). Here we found that the HbA1c was negatively correlated with Ct.Th and F_max_, and positively correlated with NE‐xLR, which revealed that hyperglycemia is responsible for compromised structural and mechanical properties and increased nonenzymatic cross‐links in the bone material of the diabetic group. We also observed elevated fAGEs content and increased NE‐xLR in the diabetic group as compared with the control group, and the NE‐xLR was negatively correlated with PYD. Indeed, the nonenzymatic cross‐links favor material rigidity by restricting the deformation (plasticity) of collagen fibers, reduce fibril stretching and sliding, and thereby reduce tissue ductility and toughness, which makes the bone more brittle.^(^ ^54^, ^55^, ^56^ ^)^ This altered matrix property facilitates microcrack generation and crack growth, and thus makes the bone more susceptible to fracture.^(^ ^57^, ^58^ ^)^ Similar to our results, Saito and colleagues^(^ ^20^ ^)^ demonstrated increased NE‐xL (pentosidine) in spontaneously diabetic WBN/Kob (Wistar Bonn Kobori) rats. Acevedo and colleagues^(^ ^24^ ^)^ showed increased AGEs content by 27% in obese UCD‐T2DM rats versus lean SD rats. Therefore, the elevated levels of fAGEs and NE‐xLR found in our study explain the material incompetence–lower toughness and ductility (PYD) in three‐point bending, and the reduced energy storage (Avg‐ED) and lower fracture resistance (IDI) at the material level (cRPI) for diabetic bone as compared with the control group.

Similar to our outbred genetically normal nonobese T2D rat model, other diabetic rodent models (spontaneous, abnormal leptin/leptin receptor signaling, diet‐induced obesity) also showed a similar trend in the three‐point bending test. The nonobese rodent model of T2D published by Saito and colleagues^(^ ^20^ ^)^ demonstrated deteriorations in the structural mechanical properties of stiffness, modulus, ultimate load, and energy absorption in the femur of spontaneously diabetic (onset of diabetes at 12 to 13 months of age) nonobese male WBN/Kob rats versus Wistar (nondiabetic) controls (kept on same diet). Zhang and colleagues^(^ ^11^ ^)^ showed a decrease in the maximum load by 21% and energy absorption by 29.7% in the femur of age‐ and sex‐matched 6‐month‐old spontaneous diabetic (onset early after birth) nonobese male Goto‐Kakizaki (GK) rats versus male Wistar rats (kept on the same diet). Although no significant difference is reported in elastic modulus in both groups, it might be because different rat strains were used for comparison.

Other than nonobese T2D rat models, there have been many obese diabetic rat models that have shown a decline in mechanical properties in the three‐point bending test. Kimura and colleagues^(^ ^59^ ^)^ demonstrated deteriorations in maximum load (44%), stiffness (28%), and energy absorption (77%) in the femur of spontaneously diabetic Torii (SDT‐fa/fa) rats (obese T2D, onset at 8 weeks of age) compared with SD rats (control animals) at 40 weeks of age (kept on the same diet). Prisby and colleagues^(^ ^13^ ^)^ observed a significant reduction in the ultimate load (NS, by 18.8%) and stiffness (17.5% and 23%) in the femur of diabetic male ZDFfa/fa (onset at 10 to 12 weeks) versus ZDF+/? (control) rats at 13 and 20 weeks of age, respectively (kept on the same diet). Reinwald and colleagues^(^ ^16^ ^)^ showed a decreased ultimate load by 30%, stiffness by 39%, and work to fracture by 36% in the femur of 33‐week‐old male diabetic ZDFfa/fa (fatty) versus ZDFfa/+ (lean control) rats. They also observed a significant reduction in the ultimate load by 19% and stiffness by 15.6% in the femur of age‐matched ZDSD compared with CDSD (Cohen diabetic Sprague‐Dawley) rats (disease onset 15 to 21 weeks of age). Gallant and colleagues ^(^ ^34^ ^)^ found decreased ultimate stress by 14.9%, modulus by 10.4%, and toughness by 50%, and postyield toughness by 67% in the femur of T2D ZDSD male rats compared with control CD male rats at 32 weeks of age (HFD given for 12 weeks, since 20 weeks of age). Reddy and colleagues^(^ ^60^ ^)^ showed reduced maximum load by 37%, bending stiffness by 38%, and energy absorption to yield and toughness by 27% and 34%, respectively, in the femur of 10‐week‐old SD rats treated with STZ (65 mg/kg body weight) for 7 weeks. All studies published so far using various T2D rat models have shown lower femoral bone strength in the diabetic group with the three‐point bending test, as reported in this study.

The advantage of our HFD‐fed/low‐dose STZ‐treated model is that it is neither inbred nor genetically determined, is easily available, and is relatively inexpensive. This model also shows the abnormalities in lipid metabolism (evidenced by increased plasma triglyceride and plasma total cholesterol levels), which contribute to various cardiovascular complications, as in the case of human T2D patients. The presented model (HFD‐fed/low‐dose STZ) can develop diabetes in both male and female rat models,^(^ ^23^, ^61^, ^62^ ^)^ whereas in ZDF and ZDSD rat models only male rats are prone to become diabetic,^(^ ^16^, ^21^, ^22^, ^23^ ^)^ and the accessibility of animals and/or expense also tend to limit their utility.^(^ ^23^, ^62^ ^)^

The main limitation of the presented model is that the use of a (low‐dose) chemical treatment (STZ), which causes a partial loss of pancreatic beta cells by direct cytotoxic action (unlike in humans). It is unique and different from other combination rat models because the dose of STZ selected causes diabetes only in HFD‐fed insulin‐resistant rats, whereas it fails to induce the same in normal control rats resembling the situation in humans with risk factors of insulin resistance to be more prone to develop type 2 diabetes than others without them. If high dose of STZ (>50 mg kg^−1^) is used then it causes direct insulin deficiency rather than insulin resistance and produces a drastic reduction in body weight. Hence, it depicts symptoms and characteristics typically more of human type 1 rather than type 2 diabetes.^(^ ^25^, ^60^ ^)^ If STZ treatment is not used, then the feeding of HFD alone requires a long time, as well as no hyperglycemia—which develops upon simple dietary treatment in genetically normal animals.^(^ ^25^ ^)^ Thus, the combination of HFD‐fed/low‐dose STZ treatment was adopted to develop T2D, which simulated the condition of mild hyperglycemia (condition similar to prediabetes) caused by insulin resistance (because of HFD for 4 weeks, hyperinsulinemia) and further developed hyperglycemia caused by low‐dose STZ treatment on HFD‐fed insulin‐resistant animals (hypoinsulinemia). This condition closely simulates the phenotype of nonobese Asian T2D as they have less insulin resistance (not to the same extent as in obese patients) and disproportionally reduced insulin secretion, as compared with obese patients with T2D (white patients). Importantly, nonobese patients with T2D have a similar increased risk of cardiovascular disease as obese T2D patients.^(^ ^7^, ^63^ ^)^

In summary, the combination of a HFD‐fed/low‐dose STZ‐treated T2D nonobese rat model was developed by using genetically normal outbred female SD rats to simulate the natural history and metabolic characteristics of late‐stage of T2D in nonobese young (and/or adolescent) T2D patients (Asians). This study also showed that the NE‐xLR is elevated in the T2D group, and strongly and negatively correlated with PYD, which directly explains the bone fragility. Along with the reduced modulus (NI) and mineral‐to‐matrix ratio (FTIR), increased IDI (cRPI) and wider mineral crystallite size (XRD) in the T2D group evidenced that the composition of diabetic bone changed: It became weaker and tended to easily fracture.

In conclusion, HFD‐fed/low‐dose STZ‐treated T2D nonobese rat model can simulate the natural history and metabolic characteristics of the nonobese young (and/or adolescent) Asian T2D patients. This study also showed that 8‐week‐long, persistent hyperglycemia affects the femoral bone quality at various organization levels. Notably, the increased nonenzymatic cross‐links result in compromised mechanical performance and diminished bone strength in T2D. Furthermore, the clear understanding of this model and the impact of diabetes on mineral and collagen quality could be helpful in designing specific treatment strategies for nonobese diabetic patients.

## Disclosures

Praveer Sihota, Ram Naresh Yadav, Sumathi Poleboina, Vishwajeet Mehandia, Sanjay Kumar Bhadada, Kulbhushan Tikoo, and Navin Kumar declare that they have no conflict of interest.

## Supporting information


**Fig. S1.** These XY plots show interrelationship between different parameters.

## Data Availability

The datasets generated during and/or analyzed during the current study are available from the corresponding author on reasonable request.
