# Supplementary figures and images for: Development of HFD‐Fed/Low‐Dose STZ‐Treated Female Sprague‐Dawley Rat Model to Investigate Diabetic Bone Fragility at Different Organization Levels
Source: JBMR Plus. 2020 Oct 20;4(10):e10379. doi: 10.1002/jbm4.10379 (PMC7574700; doi:10.1002/jbm4.10379)

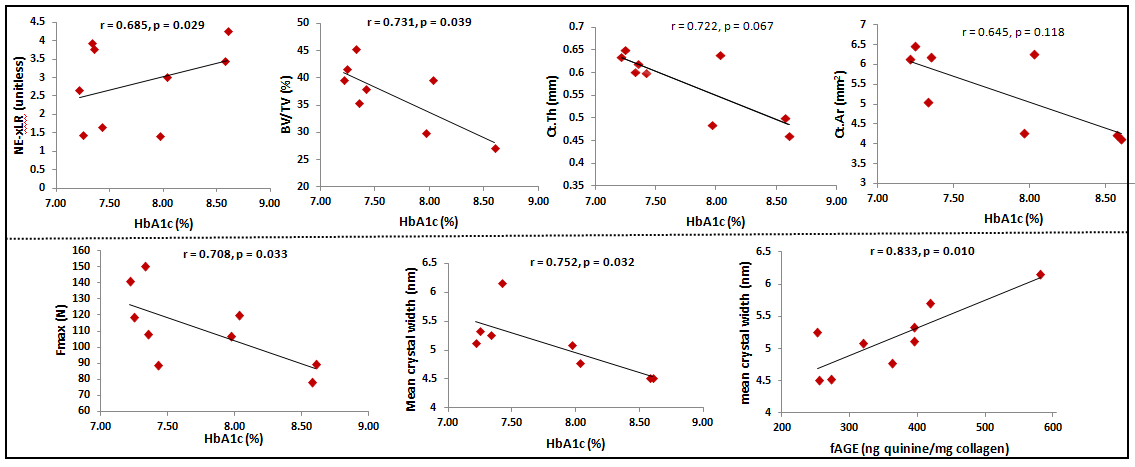


**Figure S1:** This XY plots showing interrelationship between different parameters

Supplement: Supplementary file 1 — Fig. S1. These XY plots show interrelationship between different parameters. [file JBM4-4-e10379-s001.docx]
